# Pediatric Primary Care Providers’ Perspectives on Telehealth Platforms to Support Care for Transgender and Gender-Diverse Youths: Exploratory Qualitative Study

**DOI:** 10.2196/39118

**Published:** 2023-01-31

**Authors:** Gina M Sequeira, Nicole F Kahn, Kevin M Bocek, Taraneh Shafii, Peter G Asante, Dimitri A Christakis, Wanda Pratt, Laura P Richardson

**Affiliations:** 1 Seattle Children's Research Institute Seattle, WA United States; 2 Department of Pediatrics University of Washington Seattle, WA United States; 3 Information School University of Washington Seattle, WA United States

**Keywords:** telehealth, specialist-to-PCP consultation, primary care provider, gender-affirming care, transgender youths

## Abstract

**Background:**

Access to gender-affirming care services for transgender and gender-diverse youths is limited, in part because this care is currently provided primarily by specialists. Telehealth platforms that enable primary care providers (PCPs) to receive education from and consult specialists may help improve the access to such services. However, little is known about PCPs’ preferences regarding receiving this support.

**Objective:**

This study aimed to explore pediatric PCPs’ perspectives regarding optimal ways to provide telehealth-based support to facilitate gender-affirming care provision in the primary care setting.

**Methods:**

PCPs who had previously requested support from the Seattle Children’s Gender Clinic were recruited to participate in semistructured, 1-hour web-based interviews. Overall, 3 specialist-to-PCP telehealth modalities (tele-education, electronic consultation, and telephonic consultation) were described, and the participants were invited to share their perspectives on the benefits and drawbacks of each modality, which modality would be the most effective, and the most important characteristics or outcomes of a successful platform. Interviews were transcribed and analyzed using a reflexive thematic analysis framework.

**Results:**

The interviews were completed with 15 pediatric PCPs. The benefits of the tele-education platform were developing a network with other PCPs to facilitate shared learning, receiving comprehensive didactic and case-based education, having scheduled education sessions, and increasing provider confidence. The drawbacks were requiring a substantial time commitment and not allowing for real-time, patient-specific consultation. The benefits of the electronic consultation platform were convenient and efficient communication, documentation in the electronic health record, the ability to bill for provider time, and sufficient time to synthesize information. The drawbacks of this platform were electronic health record–related difficulties, text-based communication challenges, inability to receive an answer in real time, forced conversations with patients about billing, and limitations for providers who lack baseline knowledge. With respect to telephonic consultation, the benefits were having a dialogue with a specialist, receiving compensation for PCP’s time, and helping with high acuity or complex cases. The drawbacks were challenges associated with using the phone for communication, the limited expertise of the responding providers, and the lack of utility for nonemergent issues. Regarding the most effective platform, the responses were mixed, with 27% (4/15) preferring the electronic consultation, 27% (4/15) preferring tele-education, 20% (3/15) preferring telephonic consultation, and the remaining 27% (4/15) suggesting a hybrid of the 3 models.

**Conclusions:**

A diverse suite of telehealth-based training and consultation services must be developed to meet the needs of PCPs with different levels of experience and training in gender-affirming care. Beyond the widely used telephonic consultation model, electronic consultation and tele-education may provide important alternative training and consultation opportunities to facilitate greater PCP independence and promote wider access to gender-affirming care.

## Introduction

### Background

As the population of youths who identify as transgender and gender-diverse (TGD) youths continues to grow [1], the need for gender-affirming care in pediatric settings substantially exceeds availability, leaving many TGD youths who are interested in receiving this care without access to it [2]. Given that the existing research suggests that access to gender-affirming care during adolescence is associated with improved mental health outcomes [3-8], increasing the availability of this care for TGD youths is critical. Currently, the provision of gender-affirming care is largely limited to specialty clinics located within pediatric hospital systems in large urban areas [9-15]. One way to improve the access and remove the barriers to gender-affirming care is by providing such care in the primary care setting. However, only a few pediatric primary care providers (PCPs) have received training in gender-affirming care [13-15], and many are unaware of how to create affirming environments and discuss treatment options available to TGD youths. Therefore, pediatric PCPs need opportunities to receive education from and consult gender specialists.

### Prior Work

Telehealth has the potential to meet these needs and is an umbrella term that describes both patient-to-provider audio-video visits (telemedicine) and specialist-to-PCP consultation methods, such as tele-education, electronic consultation, and telephonic consultation. Tele-education platforms connect cohorts of PCPs with specialists via the web for live didactic education and case consultation sessions. This modality, which has been used to facilitate gender-affirming care provision to TGD adults [16,17], increases provider knowledge and improves PCP’s clinical confidence [18]. Electronic consultation uses store-and-forward electronic dialogue to provide patient-specific, specialist-to-PCP consultation. This modality, which has also shown great promise in facilitating gender-affirming care provision in primary care for TGD adults [19-22], has led to increased provider knowledge along with decreased barriers to accessing specialty care [23-25]. Finally, telephonic consultation, which is the most common of these consultation models, typically involves PCPs calling an on-call specialist to discuss a specific case over the telephone [26]. These informal consultations, often referred to as “curbside” consultations, have raised concerns among specialists regarding the quality of care, patient safety, documentation, and compensation [26,27].

### Goal of This Study

Given the increasing prevalence of gender diversity [1], the inaccessibility of pediatric gender-affirming care among many youths [2,12], and the lack of training among pediatric PCPs, we must develop specialist-to-PCP telehealth platforms to guide PCPs in providing gender-affirming care. These platforms are critical because they can provide remote training and consultation, thus broadening the reach of pediatric gender-affirming care services to diverse and underresourced settings and populations. To our knowledge, no prior studies have been conducted with pediatric PCPs about how best to use specialist-to-PCP telehealth platforms, such as tele-education, telephonic consultation, and electronic consultation, to support them in providing gender-affirming care to TGD youth. Thus, the purpose of this qualitative study was to explore pediatric PCPs’ perspectives regarding optimal ways to provide telehealth-based support to facilitate gender-affirming care provision in pediatric primary care settings.

## Methods

### Recruitment

Potential participants were identified from a list of community pediatric providers across Washington, Wyoming, Alaska, Montana, and Idaho who had either previously (1) called or emailed the Seattle Children’s Gender Clinic (SCGC) team for support with a patient management question or (2) attended continuing medical education training provided by SCGC in the 2 years before recruitment. The participants were recruited via email by a member of the research team who provided an overview of the proposed study. Invitations to participate in the study were sent to 69 potential participants via email. Of these 69 individuals, 20 (33%) completed a screening survey that was used to determine study eligibility. Eligibility criteria included the following: (1) currently in practice delivering ambulatory primary care to patients aged <18 years, and (2) able to complete an hour-long audio-video interview via Zoom (Zoom Video Communications, Inc) [28]. Of 20 individuals who completed the survey, 15 agreed to participate in a semistructured interview with a member of the research team trained in qualitative research.

### Data Collection

Demographic information was collected from the screening survey and included participants’ age, race, ethnicity, gender identity, years in practice, and practice location (urban, rural, or suburban); the number of TGD youths they have seen in their practice; and the number of patients they have referred to SCGC. Details regarding tele-education preferences were collected using semistructured interviews based on guides developed with input from 3 pediatric PCP stakeholders and a community advisory board of TGD youths and their parents. The interview guide consisted of 2 parts. The first part explored PCPs’ perspectives regarding their role in providing gender-affirming care and the barriers they have faced in the primary care setting. The second part of the interview presented 3 different telehealth modalities (tele-education, electronic consultation, and telephonic consultation) using standardized definitions (Multimedia Appendix 1), and each participant was asked to share their perspectives on the following: (1) the benefits and drawbacks of each modality, (2) which modality would be most effective in supporting them in providing gender-affirming care in the primary care setting, and (3) the most important characteristics or outcomes of a successful platform. The data presented in this paper are limited to those collected in the second portion of the interview.

### Data Analysis

Interview transcripts were automatically generated via Zoom with an embedded transcription software and were cleaned and corrected by 2 trained research coordinators. Then the transcripts were independently coded by 2 members of the research team using a codebook consisting of 64 codes that was developed in partnership with a PCP stakeholder who is currently providing gender-affirming care to TGD youths. Themes were then iteratively generated using a reflexive thematic analysis framework [29]. Coding was performed using the qualitative analysis software Dedoose (Socio Cultural Research Consultants, LLC) [30].

### Ethics Approval

The participants provided informed consent to participate in the study and received a gift card worth US $20 for their participation. All study procedures were approved by the SCGC’s institutional review board (STUDY00002986) before recruitment.

## Results

### Participant Characteristics

Interviews were completed with 15 pediatric medical providers, including advanced practice providers, pediatricians, and family medicine physicians currently providing outpatient clinical care to youths aged <18 years. The participants represented a wide range of years in practice, with one-third (5/15, 33%) having practiced for >10 years and 40% (6/15) having practiced for <5 years. Nearly half (7/15, 47%) of the participants practiced in an urban area, whereas the remaining practiced in rural (4/15, 27%) and suburban (4/15, 27%) environments. Roughly half (7/15, 47%) of the participants indicated that they had seen >15 TGD patients, whereas one-third (5/15, 33%) indicated that they had seen ≤10 TGD patients. Finally, 40% (6/15) of the participants indicated that they had referred >5 patients to a gender clinic for care, with the remaining 60% (9/15) stating that they had referred ≤5 patients.

### Perspectives on the Proposed Telehealth Platforms

#### Tele-Education Platform

With respect to the advantages of the tele-education platform (Textbox 1), the following themes were identified: (1) developing a network with other PCPs that facilitates shared learning; (2) receiving comprehensive, didactic, and case-based learning; (3) having scheduled education sessions; and (4) increasing provider confidence in delivering gender-affirming care.

**Themes primary care providers (PCPs) identified as benefits and drawbacks of the tele-education platform.**

**Benefits**

- Theme 1: developing a network with other PCPs facilitates shared learning
  “You can develop some sense of...community and get to know other providers who are doing similar work nearby.”
  “It brings communities together. So, it breaks down the siloed walls of different institutions where we can really support each other...”
- Theme 2: receiving comprehensive, didactic, and case-based learning
  “I think the benefit being that you can get...more in-depth, education, you can get a good overview rather than you saying ‘Gosh I think I need to know more about this,’ you know somebody else can say ‘You need to know about this, and this, and this, and this,’ because I’m probably going to be missing something if I just pick it up myself...So...a more overarching education, probably be able to get more in depth...”
  “I think...even if I didn’t have a case to bring, I think I could learn. Or...if I have a patient that’s similar to that I could...learn from that and potentially implement something”
- Theme 3: education sessions occur at a scheduled time
  “Another thing would be if it’s predictable...if it’s once a month, at the same time, it’s something where people could plan their schedule around it and just have it already known that they’re going to talk about, they’re going to be available that time and they can make it.”
- Theme 4: increases provider confidence in delivering gender-affirming care
  “I think [tele-education] will break down the fear of starting gender affirming healthcare for a lot of people out there, especially if they’re able to kind of walk through things.”

**Drawbacks**

- Theme 1: requiring a significant time commitment
  1a: hard to prioritize over other training opportunities
    - “It’s hard to prioritize as a clinician. They’re often like...12 sessions...over a course of three months. And there’s a lot of requests for different ECHO trainings...so I tend to do one per year and I’m not sure if I would choose to do one on...transgender care now, but maybe historically would have.”
  1b: may not be worth the time investment for PCPs who see fewer transgender and gender-diverse patients or those in close proximity to a gender clinic
    - “I don’t know if I have enough volume in my clinic to have up to date, questions, or case studies. It’s a low volume, kind of, high acuity thing.”
    - “I think the cynical side would be in New Mexico, if you’re practicing in a rural place where...it’s just you, you are motivated to...fix that liver failure. But...when you’re practicing in [a large urban area with a gender clinic] and you’re like...I could...really invest a lot of time into doing this, or I could...just write a referral.”
  1c: would take time away from clinical care and decrease productivity
    - “It does seem like it’s more resource intense because obviously you have to take time out from your clinical practice, and you have to have support of your supervisors and there’s probably some financial impact to that, and it takes more time to get to the end result.”
  1d: difficult to schedule at a time that is convenient
    - “I think one of the drawbacks is finding a time where everybody can access it...we can’t have this three days a week, every month, just so everybody can access it. So, I think that can be a little bit difficult....scheduling...puts a lot of burden on the people who hold the [tele-education] program.”
- Theme 2: not allowing for real-time, patient-specific consultation
  “I think the main drawback would be timeliness...I would have somebody in my office today, and would have a question about treatment or something of that sort, and I would have to wait two weeks, and remember to get back with them to tell them what to do and perhaps the thing that might be needed would need to be done fairly quickly.”
  “When you do case presentations for patients there are supposed to be no identifiers. Oftentimes I really need to talk about this specific patient and what’s going on.”

Regarding networking, PCPs appreciated getting to know their peers who were doing similar work and developing relationships for future collaboration. They also felt that having a didactic component and listening to other PCPs’ case presentations were important to increase their knowledge about gender-affirming care provision:

I think one of the main benefits is feeling connected to other providers in your community or beyond your community...there can be a lot of isolation in primary care when you’re providing services that aren’t provided by everybody. So, I think that’s really awesome, the community aspect of it. And I think that hearing other people talk about their cases is really valuable...listening to my colleagues present is always something that’s really interesting to me and I feel like I learned a lot that way.

In addition, the PCPs appreciated that the tele-education sessions typically took place at a scheduled time, making it easier for them to coordinate with their clinical schedules.

The participants, particularly those who already had some training in gender-affirming care, felt that having access to tele-education would help increase their confidence in providing gender-affirming care, especially during the early stages of providing such care:

Especially in a time when you’re doing...information gathering to see if it’s something that is transferable to your clinic environment, [tele-education] can be really valuable...honestly if something like this in the beginning, had existed, I would have been very likely to take it on.

In terms of the drawbacks of the tele-education platform, the PCP-identified themes were as follows: (1) requiring a significant time commitment and (2) not allowing for real-time, patient-specific consultation (Textbox 1). Regarding time, some PCPs noted not feeling that they would be able to commit enough time to participate. This was especially true for providers who reported seeing a fewer number of TGD patients and those practicing in close proximity to a gender clinic*.* PCPs also cited concerns that participating in the tele-education platform would decrease their clinical productivity:

In a system where we are paid on productivity...me taking two hours to go to a tele-education thing is six patients that I’m not seeing, right? Which is...25% of my patient load for the day, which is 25% less pay. Right? And...it’s not really about the money, but...I’m held to a productivity standard. If I’m not meeting that...I think you would lose people. Because you either have to do it before or after work or they have to do it instead of seeing patients.

PCPs also mentioned feeling that it would be very difficult to schedule the sessions at a time that is mutually convenient for a large group of providers. Finally, PCPs noted that in comparison with electronic and telephonic consultation, the cadence with which scheduled tele-education sessions take place would limit their ability to receive support regarding patient-specific management questions.

[The tele-education session is] probably happening once a month or every other month, so if you had a case and it just happened last week, you’re now waiting two months to present this patient.

#### Electronic Consultation

With respect to the advantages of the electronic consultation model, themes were identified: (1) convenient and efficient communication, (2) documentation in the electronic health record (EHR), (3) ability to bill for provider time, and (4) sufficient time to synthesize information (Textbox 2).

**Themes primary care providers identified as the benefits and drawbacks of electronic consultation.**

**Benefits**

- Theme 1: convenient and efficient communication
  “We have patients constantly throughout the day, but we have a few minutes here and there where we can, finish up typing and talk to this person. So having electronic consultation would be amazing because I could just quickly type in my question. And then knowing that no one’s expecting an immediate response, I could go back and see some patients and could carry on with my day and then, when the consultation comes back in I can use my few minutes between the next patient and look at it. We as primary care providers seem to have like three to five minutes here and there throughout the day. We don’t have a full twenty minutes or half an hour to be on the phone conducting [a telephonic consultation]”
- Theme 2: documentation in the electronic health record (EHR)
  “So I think having the electronic record to be able to refer back to would be awesome. Because maybe the question you asked about for one patient will apply to a patient in the future, so, then you can just reference back to it, I think that’s a huge strength”
- Theme 3: ability to bill for provider time
  “We have this like psychiatrist who works with us now that I can actually e-consult, which is great...Because he needs the time for this and he’s consulting for all of us, it will be an official consult that’s billed to insurance.”
- Theme 4: sufficient time to synthesize information
  “I would probably be more likely to use a web base or electronic consultation, because sometimes you just don’t have time in clinic to say everything you need to say. And sometimes you, as a medical provider, need to like, sit down and think about it to be like ‘What is my question?’”

**Drawbacks**

- Theme 1: EHR-related difficulties
  1a: incompatibility of EHR with specialists
    - “You know, so you’ve got some people that are like on a Cerner platforms, some are on Epic, some on all scripts, some are next gen. You know, we’re still not in this place where we have standardized the utility of our electronic health records and they don’t talk to each other, so I think that that could be problematic.”
  1b: using an unfamiliar EHR to submit clinical questions
    - “When we do use Epic with the other clinics system...they’re always, like, ‘Where are the labs?’ And then you say I ‘I sent the labs and here they are again.’ And they’re like, ‘I still don’t see them.’”
- Theme 2: text-based communication challenges
  2a: feels impersonal
    - “Maybe just that it’s less personalized...you don’t get to see a face on the telephone but somebody just talking to a voice... especially if you’re anxious about your care, you want reassurance that you did the right thing.”
  2b: difficulties in relaying the uniqueness of a patient
    - “You lose the sort of nuances of the, of the patient and...to think about if you knew a little bit more about...the background of the patient or the story.”
  2c: miscommunications may occur
    - “It’s nice that things are documented, but sometimes things are missed in the documentation. And so, you’re making clinical decisions or clinical consultation suggestions based off of someone’s assessment that may or may not be correct.”
  2d: does not provide opportunities for back-and-forth dialogue
    - “So I think that that sort of in-time back and forth and counseling can be really valuable...as opposed to...the written word”
  2e: limits opportunities for network building
    - “You miss out on some of the networking, like, some of the personal and interpersonal dialogue that sometimes helps relationships grow, or trust grow.”
- Theme 3: not receiving an answer in real time
  “Having a real time conversation, [is], usually quicker to come up with a, with a recommendation rather than again, having to wait 24 to 48 hours for the, for the [electronic consultation].”
- Theme 4: forced conversations with patients about billing
  “The thing that was uncomfortable for me initially, and I’ve done a few [electronic consultations] with [a psychiatrist] now is that I, as the doc, have to talk to the family about how this is going to be billed to their insurance.”
- Theme 5: may be less helpful for providers who lack baseline knowledge regarding gender-affirming care
  “I probably wouldn’t really use [the electronic consultation] right now, because...I’m not serving that many patients, but once I had more of a baseline education and I have more of a population that I’m serving it could be very helpful.”

PCPs noted that unlike telephonic consultation, electronic consultation gave them the flexibility to submit the consultation question and review the response at times that were convenient for them, which was especially helpful in ensuring that the consultation did not detract from the existing patient care responsibilities. PCPs also noted the benefit of receiving timely specialist recommendations in writing, which is not often possible with telephonic consultations. Similarly, the participants found the documentation of both their consultation and the specialist’s response in the EHR to allow them to refer back to it in the future, should a similar question arise for another patient, to be particularly helpful. In addition, a few providers noted that the electronic consultation had the potential to allow both themselves and the specialist receiving the consult to be reimbursed for their time, which is not possible with telephonic consultations. Finally, some PCPs noted feeling that the act of submitting an electronic consultation would help them to better communicate their clinical questions to the specialist:

I think that sometimes being able to put it down and refine it, like, ‘No, no, this is my question, and this is my patient,’ before you send it off has significant value, because then it helps you sort of integrate and synthesize before sending it off.

Regarding the drawbacks of electronic consultations, five themes emerged: (1) EHR-related difficulties, including EHR incompatibility and unfamiliarity; (2) text-based communication challenges; (3) not receiving an answer in real time; (4) forced conversations with patients about billing; and (5) difficulties for providers who lack baseline knowledge regarding gender-affirming care (Textbox 2). Multiple PCPs cited concerns about being unfamiliar with the EHR used by specialists in their area and that making an electronic consultation system available only to those who use a specific EHR would make it inaccessible for many PCPs*.* In addition, specific concerns arose about the text-based electronic consultation communication, which some felt could feel impersonal, make it challenging to relay the specific nuances of a case, or lead to miscommunications between providers. The participants also discussed concerns that electronic consultation may not provide opportunities to engage in back-and-forth dialogue with a specialist, as opposed to telephonic consultation, and regarding limited opportunities for networking with other providers as would be possible with tele-education. Another concern reported by PCPs regarding electronic consultation was not having the ability to receive an answer to their clinical question in real time as they would be able to do with telephonic consultation*.* Finally, some providers expressed discomfort with the idea of having to inform patients and families that they would be billed for the electronic consultation. This was illustrated well by a provider who had previously used an electronic consultation platform for psychiatry:

I hate talking about money, right? I just want to take care of patients. So I expected [talking about billing for the electronic consultation with a psychiatrist] to be a very uncomfortable conversation where I say... “You know I can reach out to our pediatric psychiatrist, but this is a special consult and it will be billed to your insurance.” And I just felt kind of gross and icky, it’s almost like the family...feels like they have to say yes.

Finally, a few PCPs noted that they felt that electronic consultation would be most useful if they had strong foundational knowledge regarding gender-affirming care, which they could receive through other continuing medical education, such as the tele-education platform.

#### Telephonic Consultation

With respect to the advantages of telephonic consultation, three themes emerged (Textbox 3): (1) having a dialogue with a specialist, (2) receiving compensation for PCPs’ time, and (3) helping with acuity or complex cases.

**Themes primary care providers identified as the benefits and drawbacks of telephonic consultation.**

**Benefits**

- Theme 1: having a dialogue with a specialist
  1a: can ask additional clarifying questions
    - “Talking with someone over the phone, sometimes it’s beneficial because they’ll ask follow-up questions that you didn’t ask that can be a learning tool, but then also identify, maybe, some blind spots that maybe should be identified before people provide a specific answer, which I think is somewhat of a safety net for catching some of the clinical biases that we might have in medical decision making.”
  1b: can relay nuances of the patient’s situation
    - “I think the obvious benefits again are timeliness and being able to sort of convey the nuances of the story, or the patient. I think that having a conversation is better than a template when you’re talking about patients.”
  1c: already comfortable using this modality
    - “I think one of the benefits is it’s a model we’re familiar with and we already do it, and so it seems pretty easy to be able to, you know, call the Children’s provider to provider line, and now I can ask for a gender specialist, instead of just an endocrinologist”
  1d: receive a response in real time
    - “When we’re in conversation with families, we can let them know, ‘Hey, I don’t have an answer to your question right now, but I know who to call, and I know that they’re going to get back to me by five o’clock and then, therefore, I will get back to you today or tomorrow morning.’”
- Theme 2: receiving compensation for primary care providers’ time
  “As our coders and billers have told us...if we do the consult the same day that’s part of our coding to have for the visit, and so it could be, you know the charge can be captured in that sense as well.”
- Theme 3: helping with high acuity or complex cases
  “I think this one would be better for those more, like you said, life and death situations. Or more severe. Like, I don’t want to, maybe they’re not like actively suicidal and I don’t need to send them to the emergency department, but, like, I’m very worried about them and I don’t want to wait 24-48 hours to hear back.”

**Drawbacks**

- Theme 1: challenges with using the phone for communication
  1a: the timing of callbacks is unpredictable and may be inconvenient
    - “It is hard when you call and [the specialist] is going to call back at the end of the day, with time zone differences. I mean, I’m not always still at work, and then if I have my cell phone, it feels like I’m on call because I want to be respectful of [the specialists’] professional time. But sometimes it’s really more disruptive, because I’m not in front of a chart or things when I get the callback. Like, out walking the dog or with kids, just other responsibilities.”
    - “If I’m seeing 24 patients in a day [and] you put in the stress of...Children’s is going to call me back and the MA is going to pull me from the room and the family’s pissed because I was already 25 minutes late for them. Yeah now I’m getting pulled out and...am stressed about the backup that’s happening.”
  1b: no visual record of specialists’ recommendations
    - “Sometimes over the phone, you are scrambling to write some sort of notes or maybe write down the number...”
  1c: difficulty in relaying necessary data
    - “I think another drawback, if there’s a way to be able to send this stuff, like, electronically, you know, like labs and things like that. If the person wanted to see them, you can just imagine, like, rattling them off to the poor person trying to help you and I’m like, ‘hold on,’ you know? So, having that visual component...would be missed in the telephone one.”
  1d: phone calls can be intimidating and awkward
    - “It’s a little intimidating to call somebody even if they’re...super nice. It can be a little, like, they’re going to think...I’m dumb, and, you know, kind of a...med student kind of feel, you know?”
- Theme 2: limited expertise of the responding providers
  “It would be nice to have a direct line to the gender clinic, so I know that the provider that I’m, that I’m paging is specifically that.”
  “Sometimes I got people and they were like ‘Oh, I don’t know that, let me get this provider to call you back,’ and then so it ended up resulting sometimes in a couple of phone calls.”
- Theme 3: lack of utility for nonemergent issues
  “I have to say...I try to be cautious about paging...just because I feel like [specialists] are so busy, right? And I think most of the gender stuff is not urgent or emergent...not even end of day kind of questions and so, although I like...having the ability to [use telephonic consultation]...I don’t know that I would use it.”

More specifically, PCPs felt that telephonic consultation allowed them to ask clarifying questions and convey subtleties of the patient’s case, which may be difficult to communicate via text-based methods such as electronic consultation. In addition, PCPs reported that the timeliness of the consultation (within 24 hours) was very reassuring and allowed them to provide a timely response to a patient or their family instead of waiting days for an electronic consultation or even weeks for tele-education. Finally, PCPs noted increased comfort in using telephonic consultation platforms, given that it is a model that many had previously used*.*

With respect to the drawbacks of telephonic consultation, 3 themes emerged (Textbox 3): the (1) challenges associated with using the phone for communication, (2) limited expertise of the responding providers, and (3) lack of utility for nonemergent issues. PCPs cited facing multiple logistical challenges, leading to frustration with phone-based communication, including receiving return phone calls at inconvenient times:

[We have] no idea what window the person is going to call us back in and so if we’re in the middle of a very difficult discussion with a family in a room having someone come and knock on the door and say ‘you have a phone call’ is very disruptive.

They also reported difficulties with not having a specialist’s recommendations documented in writing and challenges relaying necessary data (eg, laboratories) accurately over the phone.

Some PCPs noted that they found it intimidating or awkward to make calls to specialists, particularly to individuals with whom they did not have a relationship*.* Similarly, they found it cumbersome not to have a direct line of communication with a specialist who has specific experience in gender care:

I’ve gotten...an adolescent medicine provider who’s more specialized in something else, like eating disorders, or menorrhagia...and then it’s a lot of back and forth, you know? Or it’s like, ‘Oh, let me go talk to my attending about that,’ and then they...go and then they come back, or call me again later and I’m in the room.

Some PCPs also reported feeling that most consultation questions that arose regarding their TGD patients did not feel urgent or time sensitive enough to warrant a same-day response:

I usually text or email or do something like that...Just because I didn’t need the answer right away. And some of it was to...solidify knowledge...and some of it was patient care; then I would call back the family in the next day or two or the patient in the next day or two.

### Most Effective Platform

PCP perspectives regarding which platform would be most effective in supporting them in delivering gender-affirming care in the pediatric primary care setting were relatively mixed, with nearly equivalent numbers of providers preferring tele-education, electronic consultation, and telephonic consultation. In addition, multiple PCPs indicated a preference for using a hybrid of the 3 models, citing that a single platform alone may not be sufficient to support them in delivering gender-affirming care (Table 1).

**Table 1:** Primary care providers’ preferred modality to provide support in delivering gender-affirming care in the general pediatric setting (N=15).

Preferred platform	Participants, n (%)	Characteristics of a successful platform	Representative quotes
Telephonic consultation	3 (20)	Nonjudgmental approach and timeliness	“The most useful...would probably be the telephone consultation. I think I would use it the most, and I think it would...have the most impact.”
Tele-education	4 (27)	Sense of community, practicality, ability to engage users, and comprehensiveness	“Probably the ECHO program because it’s a sustainable teaching method whereas the e-consult and the telephone, there may be some teaching involved but it’s essentially just giving you the answer. Which then tells you how to help this one person, but it may help you with a few others, but it’s really, just, very, it’s very individualized for the person in front of you. The ECHO program, not to use analogies too much, but it’s sort of the, you know, you give the person a fish, you feed him for a day, but if you teach them how to fish they’ll be able to feed themselves for the rest of their lives. So, I think, in the end, the one which is going to be the most beneficial is going to be the ECHO program.”
Electronic consultation	4 (27)	Reliability, technological accessibility, timeliness, and scalability	“I think that at the end of the day...I’d probably go with electronic consultation because it allows me as a medical provider the most flexibility. I can send that message at 7pm or 4am when I’m writing notes, as opposed to...being limited to the scope of...on your lunch hour or...within the business day.” “I think electronic consultation sort of allows for the greatest synthesis of assessment and I think that can be valuable to everyone...I need to write, send my question..., and then have that come back as a response, is probably the most valuable thing because sometimes you just need to think about it before you, sort of, ask that question.”
Hybrid	4 (27)	Flexibility, scalability, comprehensiveness, adaptability, timeliness, and integration of different modalities	“I think none of these modalities would likely be...enough on their own, right? Like, I think...in an ideal state, you would have multiple ways of communicating depending on the intensity of what’s going on. If I need to really talk to someone right now about something really intense happening with a patient right now, they’re having a severe reaction to some medicine that someone else has provided that I don’t really know about, I need to talk to them right now, right? And within 48 hours is not okay. But other things where...I have...more general questions or decisions that need to be made over some weeks, then doing them electronically is great.” “I really think it may have to be all three...the TeleECHO, I definitely don’t think will be enough regarding specific patients. The electronic consultation, you could do it that way, but I think you get more information if you had some ECHO too. And phone consultation just again, unless they’re really looking at their electronic stuff on a very...regular basis, sometimes you just gotta reach out and say...‘Do you understand what I’m saying?’...or it’s too much! Like the kid with the psych stuff, there was a lot of stuff and I just kind of wanted to say...‘These have changed, this is what’s going on, this is why this is like this now, this is what I’m thinking,’ you know? And I didn’t want to write a two page letter.”

### Characteristics of a Successful Platform

The 10 characteristics PCPs felt were most important for the success of platforms for supporting PCPs in providing gender-affirming care to TGD youths are shown in Table 2.

**Table 2:** Characteristics pediatric primary care providers (PCPs) felt were most important for the success of a platform for facilitating gender-affirming care provision in the primary care setting.

Platform characteristics	Representative quotes
Reliability	“If you guys say it’s 24-48 hours and people are not responding to me for a week, I’m going to stop. I’m going to just start using the telephone instead.”
Timeliness	“Getting things done quickly and being able to get back to either the family or the youth quickly, to figure out the next step. I find that if I let things sit for too long, things fall through the cracks.” “Where it’s not like ‘I can help you but we’re going to have to have you wait for six weeks for the next gender conference, because it was just yesterday.’ We missed it, you know?”
Ability to engage users	“Making sure to involve everybody, and you know, keep them accountable. Keeping people engaged would be huge.”
Nonjudgmental approach	“I think whenever I reach out to a specialist I really hope for someone who’s able to understand the constraints that I’m working within. And so, if I’m not able to spend more than five minutes on the phone in between patients, that specialist is okay with it being brief. Just approaching it...in a nonjudgmental way, like no question is a bad question.”
Scalability	“I think also knowing that if there were a case that anybody from the gender clinic thought the patient could use a higher level of care, if it were an option to have the patient do a consultation with somebody in the clinic would be cool.”
Practicality	“Sometimes we have [specialists] come in and...talk at a level that is, like, what they would talk to their colleagues. And I already don’t know what you’re talking about. I’m confused. And so, getting some baseline understanding that we are primary care providers...Understand that there’s going to be, terminology that you use. Acronyms that you use...that is already going to be above us. And, all providers, no matter how much we want to pretend that we don’t, we’re all a little bit proud. And so, it’s hard for us to be, like, ‘I don’t know what that means.’ And so, it would be like having that understanding [that] there’s a lot that we don’t know. And so, if they were to just start, rattling off about, ‘Here’s dosages,’ I’d be like, ‘Whoa, hang on a second. Let’s go back. Which ones are for boys and which ones are for girls?’ I think that bringing it down to the primary care level to start is important.”
Adaptability	“I think, you know, changing with the times. Because, all of this information, I feel like it’s constantly changing. I’m always reading about, new terminology, new ways that people like to be referring to, new ways that that you’re supposed to ask questions. So, changing with that and letting us know that those are changes. Because sometimes we don’t even know. We’re like, ‘Oh, is that the way it’s always been? Cool.’ And [also] teaching us, ‘Hey, this is going to be one of those things where, every single time we talk it’s going to be slightly different.’”
Comprehensiveness	“Any system has to be comprehensive if there’s going to be an ask for me and other PCPs to do more than what we’re doing right now. And to move on to [prescribing gender affirming medications] and [referring patients for gender-affirming surgeries] it is really going to take a lot of support, because we just don’t have experience [to know] when to pull the trigger.”
Integration of different modalities	“Having some sense of connectedness between them. So, if you had the capacity to say here’s my submission of my consultation, and if more information is needed, I’m happy to talk on the phone about this.”
Accessibility	“Different EMRs and making sure there’s some way to adapt to people who don’t have Epic.”

Specifically, PCPs desired platforms that provided reliable and timely consultative support as well as those that were engaging and nonjudgmental. In addition, the participants indicated that successful platforms were those that provided practical information, could scale up to having the specialist conduct a formal consultation with the patient if necessary, and could further adapt as terminology and pediatric gender-affirming care delivery evolve. Finally, PCPs desired platforms that were comprehensive, integrated, and accessible to all providers, and not just those using a specific EHR.

When asked what would make them more likely to continue using a particular platform, PCPs cited patient satisfaction, community building, and incentives for participation. First and foremost, PCPs frequently indicated that being able to improve the care they provide to TGD youths would motivate their use of these platforms:

I think the biggest thing for me is...patient satisfaction. Right? If it felt like the right thing to do for the patient and the patient was happy, grateful, thankful, whatever you want to say, right? Like, if it’s...”Oh, I can...stop your periods today.” That makes a huge difference for a 14-year-old, who...was born female and is a male, and is distressed every single month. So to be able to...help that kid would make me do it again.

Providers also felt that having opportunities to establish a sense of community with other pediatric providers interested in gender care would motivate them to continue using a platform:

Certainly, hearing from other providers having similar experiences...would make me want to go back and have another...tele-education meeting. Just to know that I’m able to glean information for my patient care from those meetings.

Finally, PCPs mentioned that being able to receive incentives, such as continuing medical education or the maintenance of certification credits, would motivate them to use a platform. This was especially true for tele-education platforms, as many participants acknowledged that such incentives could offset the significant time commitment required for participation.

## Discussion

### Principal Findings

The results of this qualitative study suggest that pediatric PCPs desire opportunities to both obtain foundational knowledge and receive timely consultative support from gender specialists regarding patient-specific concerns. The variation in these results stems from the wide variation in PCPs’ training and experience in providing gender-affirming care for TGD youths, and the current options for training and consultation in this area are quite limited [13-15]. To meet the increasing demand for gender-affirming care services for TGD youths, we must develop a diverse suite of telehealth-based training and consultation services to meet the needs of PCPs with different levels of experience and training in this area. This specialist-to-PCP support is critical for facilitating greater PCP independence in gender-affirming care provision as well as for expanding the access of TGD youths to pediatric gender-affirming care services.

Increasing requests for specialist-to-PCP telephonic consultation during the COVID-19 pandemic [31] have led many large pediatric health systems to reconsider whether these services are (1) providing the best quality care to patients and (2) sustainable for pediatric specialists [32,33]. Although informal telephonic or “curbside” consultations remain the most common form of pediatric specialist-to-PCP telehealth support, our findings suggest that it may not be the ideal modality to support PCPs in providing pediatric gender-affirming care. Although our findings indicate that PCPs perceived telephonic consultation as having some important benefits, such as the timeliness of response and wider accessibility, they also noted many drawbacks. These drawbacks, which include limited compensation for consultation services, raise concerns about the sustainability of telephonic consultation systems and indicate a need to develop new modalities to provide specialist-to-PCP support.

Consequently, several providers in our study expressed a desire for an electronic consultation platform to support the provision of gender-affirming care in the pediatric primary care setting. This modality may be particularly useful in overcoming some of the barriers that exist with telephonic consultation systems, including the lack of written documentation, inconvenience of receiving unscheduled phone calls, difficulty in exchanging laboratory data, and lack of PCP and specialist compensation. In particular, electronic consultation may be a helpful modality to increase the capacity of PCPs to submit nonurgent questions to support their TGD patients and to ensure that questions are routed directly to providers with expertise in gender-affirming care. Electronic consultation may also increase the capacity of PCPs and specialists to exchange comprehensive and patient-specific information, review objective data, and document recommendations in writing to facilitate the provision of ongoing care.

Despite these benefits, there are some challenges to developing specialist-to-PCP electronic consultation platforms. Several PCPs indicated that they desired an electronic consultation platform within the EHR used in their practice because of both its convenience and their familiarity with its functionality. However, this remains logistically challenging given the heterogeneity of EHRs used by pediatric PCPs across the United States and the reluctance of EHR vendors to adopt sustainable medical applications, reusable technologies application programming in accordance with defined standards for Fast Healthcare Interoperability Resources [2,34]. This is likely why many of the existing specialist-to-PCP electronic consultation platforms, such as those used by the Veteran Affairs health system [16,17,21], are available to only PCPs who work within the same health system as that of the specialists providing the consultation. Ensuring that pediatric gender-affirming care provision is accessible and equitable will require the use of modalities that are widely accessible to providers in diverse clinical practice settings [35].

Tele-education may also be a particularly useful modality for PCPs whose practices are located farther from a pediatric gender specialist or who are seeing an increasing number of TGD patients [18]. Regarding geography, providers located farther from pediatric multidisciplinary gender clinics may be more inclined to dedicate time to formal education sessions, as they serve patients who face additional access- and travel-related barriers to receiving specialty care. Given that this platform would provide them with an opportunity to receive more comprehensive foundational knowledge, providers in remote areas may be more willing to invest time upfront, despite the clinical sacrifices, knowing that it would facilitate care for their patients. Finally, patient volume, specifically, the number of TGD youths seen in their practice, may impact their interest in a tele-education platform. On the one hand, the increasing number of TGD patients may encourage PCPs to gain more formal experience working with this population; alternatively, a PCP who already sees many TGD youths in their practice could be more inclined to use telephonic or electronic consultation, as they are more likely to have developed foundational knowledge and skills through practice.

### Limitations

This study should be interpreted within the context of the following limitations. Although diversity existed with respect to participants’ primary practice locations and years in practice and the number of patients they referred to a gender clinic, the participants were relatively homogeneous with respect to gender identity and race and ethnicity. In addition, both our response rate and decision to recruit PCPs who had previously sought support may limit the generalizability of our findings. Although the providers in our study may be more likely to use a telehealth platform for support in providing gender-affirming care than those who have not sought out this support, we are confident that these data reflect the perspectives of PCPs who are the most motivated to use a telehealth platform for support in providing care for TGD youths in the primary care setting. Furthermore, although our interview guide was intentionally designed to obtain PCPs’ perspectives about both the advantages and disadvantages of each modality, it is possible that social desirability bias affected our findings. Finally, given that specialist-to-PCP telephonic consultation systems are currently in use in many pediatric hospital systems, it is likely that the PCPs in our study had more experience using this modality than electronic consultation or tele-education, which could, in turn, have affected their responses.

### Conclusions

In summary, our findings suggest that beyond the current telephonic consultation model, electronic consultation and tele-education may provide important alternative training and consultation platforms to support pediatric PCPs in providing gender-affirming care to TGD youths. Improving specialist-to-PCP support in these ways is critical for facilitating greater PCP independence in gender-affirming care provision and promoting widespread access to pediatric gender-affirming care services for TGD youths.

## Appendix

Multimedia Appendix 1 Definitions used to describe each telehealth platform to the interview participants.

## Abbreviations

EHR: electronic health record
PCP: primary care provider
SCGC: Seattle Children’s Gender Clinic
TGD: transgender and gender-diverse
