# Supplementary material for: Pediatric Primary Care Providers’ Perspectives on Telehealth Platforms to Support Care for Transgender and Gender-Diverse Youths: Exploratory Qualitative Study
Source: JMIR Hum Factors. 2023 Jan 31;10:e39118. doi: 10.2196/39118 (PMC9929719; doi:10.2196/39118)
Supplement: Multimedia Appendix 1 [file humanfactors_v10i1e39118_app1.docx]

**Appendix 1**

Definitions used to describe each telehealth platform to interview participants.

| Telehealth modality | Definition used to describe telehealth modality with participants |
| --- | --- |
| *Tele-education* | *Tele-education (also known as ECHO) is a model that virtually connects groups of community providers with a team of specialists for regular, real-time collaborative sessions that incorporate both didactic education and opportunities for consultation. For example, a tele-education platform could involve a small group of community PCPs meeting virtually on a monthly basis with a team of gender clinic providers to receive education about gender care and present cases for consultation.* |
| *Electronic consultation* | *Electronic consultation uses a shared electronic health record (EHR) or other web-based platforms to provide opportunities for timely PCP to specialist communication and consultation. For example, a PCP could enter a patient-specific management question via a pre-populated consultation template in EPIC that would be answered by a gender clinic provider via EPIC message within 24-48 hours.* |
| *Telephonic consultation* | *Telephonic consultation involves consultation between PCPs and specialists to discuss patient management via telephone. As an example, a PCP could call an on-call line and request a callback later that day from a gender clinic provider to discuss a specific gender-related management question over the phone.* |
